# Gene action, genetic variation, and GWAS: A user-friendly web tool

**DOI:** 10.1371/journal.pgen.1009548

**Published:** 2021-05-20

**Authors:** Valentin Hivert, Naomi R. Wray, Peter M. Visscher

**Affiliations:** 1 Institute for Molecular Bioscience, The University of Queensland, Brisbane, Queensland, Australia; 2 Queensland Brain Institute, The University of Queensland, Brisbane, Queensland, Australia; University of California Los Angeles, UNITED STATES

## Abstract

Fisher’s partitioning of genotypic values and genetic variance is highly relevant in the current era of genome-wide association studies (GWASs). However, despite being more than a century old, a number of persistent misconceptions related to nonadditive genetic effects remain. We developed a user-friendly web tool, the Falconer ShinyApp, to show how the combination of gene action and allele frequencies at causal loci translate to genetic variance and genetic variance components for a complex trait. The app can be used to demonstrate the relationship between a SNP effect size estimated from GWAS and the variation the SNP generates in the population, i.e., how locus-specific effects lead to individual differences in traits. In addition, it can also be used to demonstrate how within and between locus interactions (dominance and epistasis, respectively) usually do not lead to a large amount of nonadditive variance relative to additive variance, and therefore, that these interactions usually do not explain individual differences in a population.

## Introduction

It is generally agreed that the theoretical foundation of quantitative (complex) trait genetics was the 1918 paper from RA Fisher [1]. Fisher showed how the effect sizes and gene action within and between trait loci could be summarized as genetic variance components and that these genetic variances could be estimated from the resemblance between relatives, without knowing anything about what we now term “genetic architecture,” i.e., the number of causal loci and the joint distribution of allele frequency and effect sizes at those loci. Douglas Falconer popularized quantitative genetics in 1960, by providing an introduction to its general concepts with the first edition of his now famous book “Introduction to Quantitative Genetics” [2]. Falconer used the analysis of variance methodology of Fisher [1], but provided simpler and more intuitive notation and derivations. The book was a huge success, with 3 subsequent editions, the last one being Falconer and Mackay [3]. Extension of the concepts is provided in the advanced text book of Lynch and Walsh [4].

Although Fisher’s variance decomposition is more than a century old, it is highly relevant today because we now have the genomic tools to identify individual trait loci, in particular through the experimental design of the genome-wide association study (GWAS). GWAS essentially uses Fisher’s [1] method of partitioning genotypic values by performing a linear regression of the trait on SNP allelic dosage [5]. In just over a decade, hundreds of thousands of genetic variants have been associated to traits in both model and non-model species. In human applications, as of January 29, 2021, the GWAS Catalog [6] contained 4,845 publications and 246,178 associations from data mapped to Genome Assembly GRCh38.p13 and dbSNP Build 153.

However, we and others have found that the concepts that link gene–gene interactions (dominance within a locus and epistasis between loci) with genetic variation and thereby individual differences in a population remain confusing and misunderstood. Here, we present an R ShinyApp for use as a tool for teaching and learning quantitative genetics. We named it the Falconer ShinyApp in memory of Douglas Falconer and his tremendous contribution to the field of quantitative genetics [7].

## Results

In the Falconer ShinyApp, we illustrate Chapters 7 and 8 of Falconer and Mackay [3] as well as Chapter 5 of Lynch and Walsh [4], through 3 different genetic models. The application illustrates the concept of average effect, nonadditive effect, and the decomposition of the total genotypic variance through the 3 models. A 1-locus model with additive and dominance effect first describes the within locus interaction effect (dominance) on the average effect and additive variance. Then, a 2-locus model with additive and additive-by-additive effect illustrates the inter-locus interaction effect. Finally, a completely general 2-locus model allows the user to specify the genotypic values and allele frequencies at the 2 loci, and the resulting total genotypic variance is then partitioned into 5 variance components (additive, dominance, additive-by-additive, additive-by-dominance, and dominance-by-dominance), using a general least squares approach [4].

### One-locus model with additive and dominance effects

We first consider a single locus model with alleles A_1_ and A_2_ at frequency *p* and 1-*p*, respectively. Under panmixia (i.e., random mating) and Hardy–Weinberg equilibrium, the expected genotype frequencies are (1−*p*)^2^,2*p*(1−*p*) and *p*^2^, for A_2_A_2_, A_1_A_2_ and A_1_A_1_, respectively. We arbitrarily assign trait values -*a*, *d*, and *a* to the 3 genotypes, *d* representing the dominance effect (within locus interaction, no interaction when *d* = 0) and 2*a* the difference between the 2 homozygotes. Under this model, the population mean is *M* = (2*p*−1)*a*+2*p*(1−*p*)*d* (see Equation 7.2 of Falconer and Mackay[3]). Fisher [1] partitioned the genotypic values and the variance due to genotypes using a linear model approach, which models the genotypic values as a function of the allelic dosage (0, 1, or 2) of genotypes and the allele frequency. Except for the case of pure additivity (*d* = 0), where there is a perfect linear relationship between genotypic value and allelic dosage, the relationship between genotypic values and allelic dosage is nonlinear. Fisher used a linear model to derive the least squares linear relationship between genotypic value and allelic dosage and thereby partition of genotypic values of traits in terms of expected values under pure additivity (called “breeding values” in the literature) and deviations from this model due to the within locus interaction (dominance). The slope of the linear regression of the trait genotype means, weighted by their frequency, on *A*_1_ allelic dosage (0, 1, or 2) is the average effect of allelic substitution at a locus (confusingly also called “additive effect” in the literature):

α=a+(1−2p)d (1)

The average effect depends both on half of the difference between the genotypic values of the 2 homozygotes (*a*) and the dominance value *d*, as does the additive variance (see below). The residuals of the linear regression are the deviations due to the within locus interaction (dominance). Fisher [1] partitioned the total genotypic variance (*V*_*G*_) of this model as

$$V_{G}=V_{A}+V_{D},$$

with *V*_*A*_ as the variance of the additive (breeding) values, which is known as the additive variance, and *V*_*D*_ as the dominance variance. *V*_*A*_ is the variance due to the regression of genotype means onto allele dosage and is given as *V*_*A*_ = 2*p*(1−*p*)*α*^2^ = *Hα*^2^, with *H* being the heterozygosity under Hardy–Weinberg equilibrium of the genotype frequencies. Notably, *V*_*A*_ includes contributions from dominance effects (through *α*, see **Eq 1**) when *p*≠0.5. Similarly, the dominance variance due to within locus interaction is the variance of the residuals of the linear regression, *V*_*D*_ = (2*p*(1−*p*)*d*)^2^ = *H*^2^*d*^2^. Therefore, the dominance variance disproportionally depends on the locus heterozygosity compared to the additive variance (*H*^2^ versus *H*) and reaches its maximum at *p* = 0.5 where dominance deviations are not captured by the linear regression and do not contribute to *α* (**Eq 1**). In summary, the additive genetic variance *V*_*A*_ is the regression variance, and *V*_*D*_ is the residual variance from a regression of genotypic mean trait value on allelic dosage.

When performing a GWAS, individual phenotypes *y* are regressed on the number *x* (*x* = 0, 1, 2) of reference alleles at a given locus, i.e., the allelic “dosage,” where the reference allele for this dosage count is arbitrarily the major or minor allele (but this arbitrary choice is reflected in the sign of the regression coefficient *β*): *y* = *μ*+*βx*+*e*, where the residuals *e* include both the nonadditive genetic effects at the locus, the genetic effects (additive and nonadditive) at other loci, and an environmental and/or chance (nongenetic) effect. The quantity of interest is the slope *β* of the model (the effect size of the locus), which is the average effect of allele substitution, hence *β* = *α*, because the expected phenotypic value of a genotype is its expected genotypic value, under the assumption of independence between genetic and environmental effects. Hence, performing a regression of phenotype on allelic dosage at, for example, a SNP marker, as routinely done in GWAS, is equivalent to estimating the average effect size, and the regression variance is the additive genetic variance due to the locus.

For noncontinuous trait and GWAS, the same model as above can be used on either the actual scale of the trait measurement or on a transformed scale. For example, for binary disease traits (*y* = 0 or *y* = 1), GWAS analysis is often performed using logistic regression, so that *β* and the regression variance are on an underlying logistic scale. Note that a nonlinear transformation between the observed (0 and 1) and underlying continuous scale implies that absence of dominance variance on one scale (for example, logic or probit) does not imply absence on the other scale.

In the Falconer ShinyApp, we let the user choose the arbitrarily assigned genotypic values by specifying *a*, *d*, and the allele frequency *p* in a predefined range. Given these values, we adapted the Table 7.2 and Fig 7.3 of Falconer and Mackay [3]. Hence, we display the population trait mean, genotypic values (i.e., trait means per genotype class, both absolute and expressed as deviation from the population mean), additive (breeding) values, and dominance deviations, as well as the linear regression of the genotypic values weighted by their frequency on the *A*_*1*_ allele dosage. Finally, we display the distribution of the additive (*V*_*A*_) and dominance (*V*_*D*_) genetic variance as well as the proportion of total genetic variance explained by the additive variance (*V*_*A*_/*V*_*G*_) as a function of the arbitrarily assigned genotypic values *a* and *d*.

**Fig 1:**
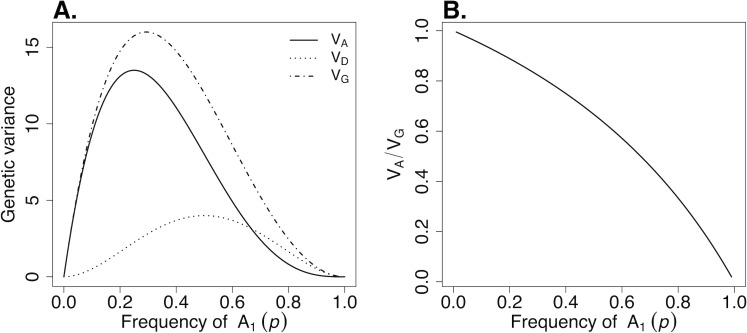
Numerical example for a single locus model with genotypic value *a* = 4 for the homozygote A_1_A_1_ and complete dominance of A_1_ (*d* = 4). Distributions of **(A)** additive (V_A_), dominance (V_D_), and total (V_G_) genotypic variance and **(B)** proportion of genotypic variance explained by additive variance (V_A_/V_G_).

In a simple numerical example, we consider a locus with complete dominance and the A_1_ allele dominant over the recessive allele A_2_. We arbitrarily assign genotypic values *a* = 4 and *d* = 4. In this example, the distributions of the additive, dominance, and total genotypic variance, as well as the ratio *V*_*A*_/*V*_*G*_, are shown in **Fig 1**. It shows that the contribution of additive variance to the total genotypic variance (*V*_*A*_/*V*_*G*_) increases when the frequency *p* of the dominant allele decreases, with *V*_*A*_/*V*_*G*_→1 for *p*→0. It also shows that *V*_*A*_ (and *V*_*G*_) is much higher when the dominant allele is at low frequency (e.g., *p* = 0.1) than *V*_*D*_ (and *V*_*G*_) when the dominant allele is at higher frequency (e.g., *p* = 0.9).

### Two-locus model with additive and additive-by-additive effects

We extend the 1-locus to a 2-locus model with additive and additive-by-additive epistatic interaction only, assuming no within loci dominance effects (*d* = 0 at both loci). We introduce a second (unlinked) locus with alleles B_1_ and B_2_ and frequencies *q* and 1*-q*, respectively. The genotypic values and frequencies of the 9 genotypes are described in **Table 1**, where *a*_AB_ is the additive-by-additive interaction effect.

**Table 1:** Genotypic values and frequencies of a 2-locus model with additive and additive-by-additive effect and no dominance at either locus.

	A_2_A_2_	A_1_A_2_	A_1_A_1_
**B**_**2**_**B**_**2**_	**−*a***_**A**_**−*a***_**B**_**+*a***_**AB**_ (1−*p*)^2^(1−*q*)^2^	**−*a***_**B**_ 2*p*(1−*p*)(1−*q*)^2^	***a***_**A**_**−*a***_**B**_**−*a***_**AB**_ *p*^2^(1−*q*)^2^
**B**_**1**_**B**_**2**_	**−*a***_**A**_ (1−*p*)^2^2*q*(1−*q*)	**0** 4*p*(1−*p*)*q*(1−*q*)	***a***_**A**_ 2*p*^2^*q*(1−*q*)
**B**_**1**_**B**_**1**_	**−*a***_**A**_**+*a***_**B**_**−*a***_**AB**_ (1−*p*)*q*^2^	***a***_**B**_ 2*p*(1−*p*)*q*^2^	***a***_**A**_**+*a***_**B**_**+*a***_**AB**_ *p*^2^*q*^2^

This model is a re-parametrization of the model described in Mäki-Tanila and Hill [8], where our population mean becomes

$$M=a_{A}(2p-1)+a_{B}(2q-1)+a_{AB}(1-2(p+q)+4pq)$$

And the average effects at the 2 loci are

$$\alpha_{A}=a_{A}+(2q-1)a_{AB}$$ (2)

$$\alpha_{B}=a_{B}+(2p-1)a_{AB}$$

Therefore, the average effect at each locus depends on half of the difference between the genotypic values of the homozygotes (*a*_A_ or *a*_B_) as well as the additive-by-additive effect and the allele frequency of the interacting locus. In this model, the total genotypic variance (*V*_*G*_) is partitioned in additive (*V*_*A*_) and additive-by-additive variance (*V*_*AA*_, the variance due to the interaction between the breeding values at the 2 loci)

$$V_{G}=V_{A}+V_{AA},$$

with the additive variance

$$V_{A}=\sum H_{i}{\alpha_{i}}^{2},$$

where *H*_*i*_ is the frequency of heterozygote genotype at locus *i* (*i* = A, B), *α*_*i*_ the average effect at locus *i*, and the additive-by-additive variance

$$V_{AA}=H_{A}H_{B}a_{AB}^{2}$$

As for the single locus model previously described, this 2-locus model illustrates how nonadditive genetic effects, here an additive-by-additive epistatic effect, enter in the average effect term as well as in the additive variance. As for dominance variance, the additive-by-additive variance disproportionally depends on the locus heterozygosity compared to the additive variance, since additive variance depends on the sum of heterozygosities at the 2 loci, whereas additive by additive variance depends on their product.

**Fig 2:**
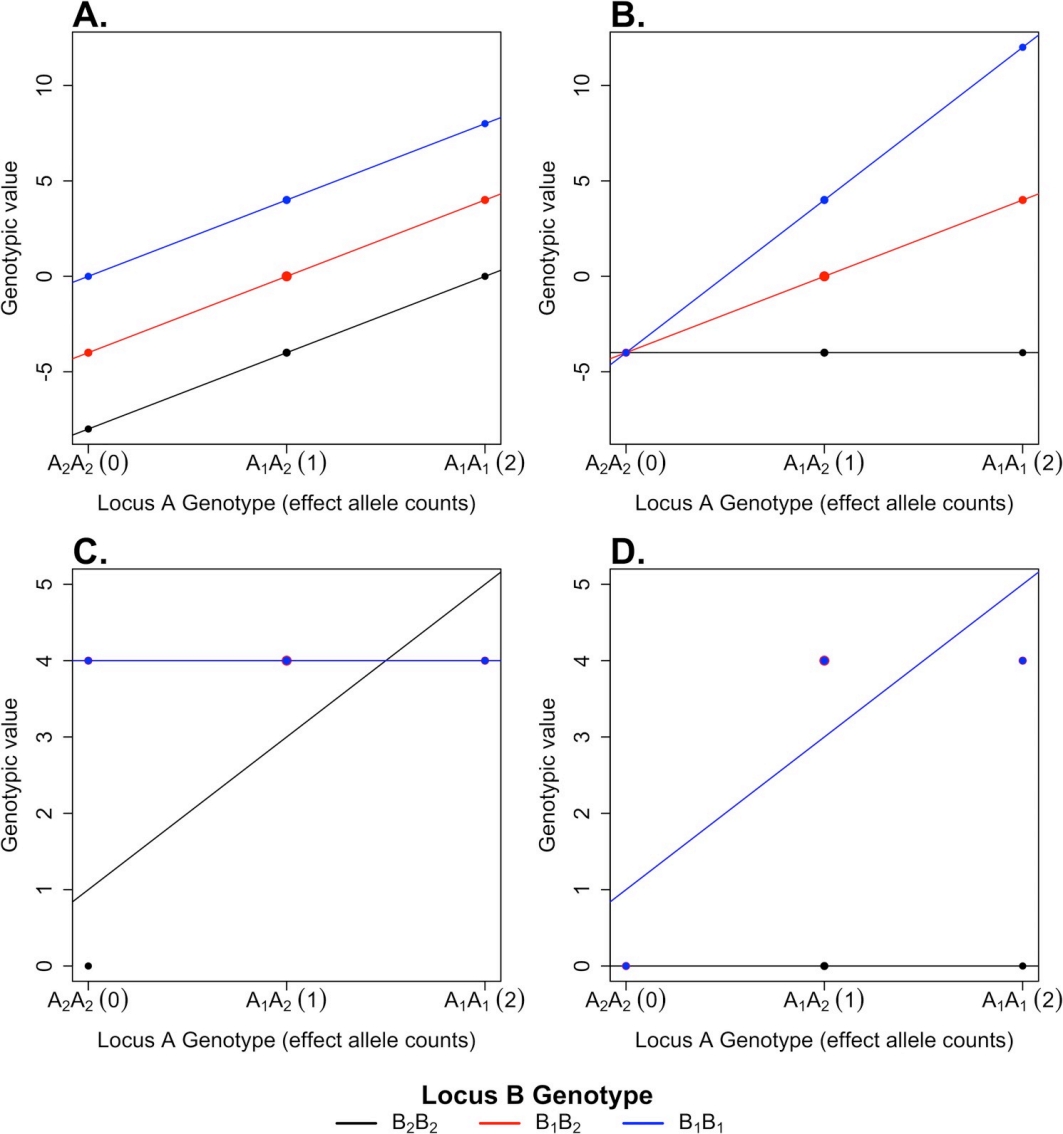
Genotypic values (closed circles) at 2 biallelic loci A and B in 4 different 2-locus models and the weighted least squares regression fits (colored lines) for each B locus genotype against A_1_ allele dosage. In **(A)**, we consider a 2-locus model with additive effects only at both loci (*a*_A_ = *a*_B_ = 4), while in **(B)**, we consider a 2-locus model with additive and additive-by-additive effects (*a*_A_ = *a*_B_ = *a*_AB_ = 4). In **(C)**, we consider a 2-locus duplicate factor model (**Table 2**), and in **(D)**, a 2-locus complementary model (**Table 3**), both with *c* = 4. The horizontal scale shows the number of A1 alleles in the genotype. The different genotypes at locus B are depicted in different colors (black for B_2_B_2_, red for B_1_B_2_, and blue for B_1_B_1_). We chose allele frequencies *p* = *q* = 0.5 in all 4 models. Each point size is weighted by its genotype frequency, and linear regression lines between the number of A_1_ allele and the genotypic values are fitted by weighted least squares for each locus B genotype background. In panels **(C)** and **(D),** the red and blue lines are overlaid.

In the Falconer ShinyApp, the user chooses the allele frequencies *p* and *q* as well as the genotypic values *a*_A_, *a*_B_, and *a*_AB_ in a predefined range. The corresponding values and frequencies for the 9 genotypes are displayed following **Table 1**, as well as the population mean M, the locus-specific average effects *α*_A_ and *α*_B_, and the different variance components. The genotypic values and linear regressions are plotted as a function of the A_1_ allelic dosage for the different backgrounds (locus B genotypes). Hence, it illustrates the departure from the additive model (*a*_AB_ = 0) when additive-by-additive interactions are present (see **Fig 2A and 2B** for a numerical example). We use a graphical representation[9] of the additive (*V*_*A*_), additive-by-additive (*V*_*AA*_), and proportion of genotypic variance explained by additive variance (*V*_*A*_/*V*_*G*_) as a function of the allele frequencies *p* and *q* given the genotypic values.

### General 2-locus model

In the previous models, we illustrated the effect of within and between loci interaction on the average effect and additive variance. Lastly, we propose a generalized 2-locus model where the user can provide all the genotypic values in an interactive table and choose the allele frequencies at the 2 (unlinked) loci (*p* and *q*). The genotypic values as well as the linear regressions are plotted as a function of the A_1_ allelic dosage for the different genotypes at locus B, and so does the linear regression of the genotypic values, weighted by their frequency on the A_1_ allele dosage. The total genotypic variance (*V*_*G*_) of this model is then partitioned in 5 components:

$$V_{G}=V_{A}+V_{D}+V_{AA}+V_{AD}+V_{DD},$$

where *V*_*AD*_ is the additive-by-dominance variance (due to the interaction between the breeding value at 1 locus and the dominance deviation at the second locus) and *V*_*DD*_ the dominance-by-dominance variance (due to the interaction between the dominance deviations at the 2 loci). Definitions and derivations of these and higher-order interaction components were given by Cockerham [10] and Kempthorne [11]. We use the least squares approach described in Lynch and Walsh [4] to calculate the different variance components and display their values.

As numerical examples, we will consider 2 classical epistatic models that involve all nonadditive components. The first example is the 2-locus duplicate factor model, such as described by Shull [12] when he studied the shape of seed capsules of shepherd’s purse plants (*Bursa bursa-pastoris*). Shull [12] found that all genotypes led to a triangular capsule, apart from the double recessive homozygote, which led to an oval shape. We consider the equivalent statistical model (**Table 2**) where genotypic values are *c* except for the double recessive homozygote which has value 0. In this highly epistatic model and as described by Hill and colleagues [13], we expect *V*_*A*_/*V*_*G*_→1 when allele frequencies *p*,*q*→0. Choosing *c* = 4 and *p* = *q* = 0.5 (**Fig 2C**), the total genotypic variance is then partitioned as

$$V_{G}=V_{A}+V_{D}+V_{AA}+V_{AD}+V_{DD}$$

0.94=0.25+0.13+0.25+0.25+0.06

Leading to *V*_*A*_/*V*_*G*_ = 0.27, in accordance with theoretical expectation [13].

**Table 2:** Genotypic values of a 2-locus duplicate factor model.

	A_2_A_2_	A_1_A_2_	A_1_A_1_
**B**_**2**_**B**_**2**_	0	*c*	*c*
**B**_**1**_**B**_**2**_	*c*	*c*	*c*
**B**_**1**_**B**_**1**_	*c*	*c*	*c*

A second classical example of epistasis is the complementary model of flower color in sweet pea (*Lathyrus odoratus*) studied by Bateson and colleagues [14]. In this species, the flower color is controlled by 2 loci, each with a dominant allele. All genotypes involving at least a recessive homozygote at 1 locus result in white flowers whereas the other genotypes lead to the dominant purple color. For this 2-locus complementary model (**Table 3**), we chose *c* = 4 and *p* = *q* = 0.5 (**Fig 2D**), resulting in a partitioning of the total genotypic variance

$$V_{G}=V_{A}+V_{D}+V_{AA}+V_{AD}+V_{DD}$$

3.93=2.25+1.12+0.25+0.25+0.06

Leading to *V*_*A*_/*V*_*G*_ = 0.57, also in accordance with theoretical expectation [13].

**Table 3:** Genotypic values of a 2-locus complementary model.

	A_2_A_2_	A_1_A_2_	A_1_A_1_
**B**_**2**_**B**_**2**_	0	0	0
**B**_**1**_**B**_**2**_	0	*c*	*c*
**B**_**1**_**B**_**1**_	0	*c*	*c*

There are special cases (at least in theory) where there is no additive variance (*α* = 0), hence where all genetic variation is nonadditive. In such cases, an additive model (e.g., as used in a standard GWAS) will fail to capture any genetic variation. That said, very little evidence for nonadditive genetic variation have been found in human traits when analyzing a broad range of phenotypes [15–17].

### Persistent misconceptions

Despite the theory being more than a century old, persistent misconceptions related to gene interactions effects remain. One common misunderstanding is that models that estimate additive effects of alleles or estimate additive variance assume that there are no gene interactions, which is not correct. Indeed, an additive model does not fit nonadditive effects explicitly; however, the average effect that is estimated is a function of nonadditive effects (both dominance and epistatic effects; see **Eqs 1 and 2**).

A second misconception is that interactions with large effect sizes should make a large contribution to the observed population variation of a complex trait. For example, a rare recessive mutation can have a large effect size but still leads to a small amount of genetic variance explained in the population. Indeed, even in the absence of dominance interactions, rare variants with large effect sizes only contribute proportionally to their frequency.

Epistatic interactions have been proposed as a potential source of genetic variation [18,19]. This leads to the third misconception that higher-order gene–gene interactions, which could, in principle, be detected by machine learning algorithms [20,21], would explain a lot of genetic variation. However, data and theory show that it is almost certainly not true and that additive variance is expected to remain the main contributor of genetic variation, even in the presence of high-order interactions [8,13]. This also means that observing a large additive variance is not inconsistent with an underlying generative model that is highly interactive.

A final misconception is that the results from model organisms on nonadditive genetic variation can be generalized to other species. Although different studies using model organisms have highlighted the importance of nonadditive genetic variation [9,22], postulating that these results translate to outbred populations, including humans, would be a mistake. The experimental designs in most model organisms are based on inbred lines, for example, segregating crosses from 2 different founder inbred strains or recombinant inbred lines (lines bred to isogenicity following an initial cross). Such designs lead to intermediate allele frequencies (typically 0.5) and thereby maximize heterozygosity and the variance attributable to nonadditive effects. However, in outbred populations, there is strong evidence of negative selection on complex traits, so that heterozygosity at causal loci is lower than its expectation under neutrality [23,24], and therefore, nonadditive variance is a priori predicted to be much smaller than in model organisms [22]. Moreover, inbreeding itself might lead to results about interactions that are not transferable to outbred populations [25].

## Conclusions

Fisher’s [1] partitioning of genotypic trait values and of phenotypic variance remains highly relevant in the GWAS era, but misunderstandings persist, especially about how nonadditive genetic effects (interactions) impact per-allele effect size and variance. We developed the Falconer ShinyApp in order to illustrate through 3 genetic models how interactions within and between loci create a departure from a linear relationship between genotype and phenotype and how they convert to average effect sizes and additive variance. We hope that it will be helpful for students and researchers learning or teaching quantitative genetics.

### Availability and implementation

The Falconer ShinyApp is accessible using web browsers at https://shiny.cnsgenomics.com/Falconer2/, and the R shiny source code is available on GitHub (https://github.com/CNSGenomics/falconer-shiny).
